# Determining the rate and reasons for specimen rejection among specimens referred for HIV viral load determination through referral linkage to Debre Tabor Comprehensive Specialized Hospital, north-central, Ethiopia,2023: Retrospective study

**DOI:** 10.1016/j.heliyon.2024.e31736

**Published:** 2024-05-22

**Authors:** Ayenew Berhan, Andargachew Almaw, Shewaneh Damtie, Yenealem Solomon, Biruk Legese, Birhanu Getie, Mulat Erkihun

**Affiliations:** Department of Medical Laboratory Science, College of Health Sciences, Debre Tabor University, Debre Tabor, Ethiopia

**Keywords:** Human immunodeficiency virus, Rejection, Viral load, Specimen, Ethiopia

## Abstract

**Background:**

The preanalytical phase encompasses the time between the clinician's test order to the sample being ready for analysis. Of all errors during the laboratory diagnostic process,70 % appeared in the pre-analytical phase. In clinical laboratories, it is crucial to ensure proper specimen collection and handling, which is essential to guarantee quality assessment, monitoring process standardization, improving performance, and ensuring patient safety. Despite this importance, no study has been conducted in the study area to investigate the rate and reasons for human immunodeficiency virus viral load sample rejection.

**Objective:**

To determine the rate of human immunodeficiency virus viral load sample rejection (number of preanalytical errors) documented during the preanalytical phase and articulate possible causes for specimen rejection.

**Material and methods:**

A retrospective study was conducted at Debre Tabor Comprehensive Specialized Hospital from January 1st to January 31, 2023. During the study period, 5950 samples were extracted from the human immunodeficiency virus viral load laboratory sample tracking log books, which were sent to the hospital for viral load testing between August 2021 to November 2022. The collected data were cleaned and entered into EPI data version 4.6 before transferred it to STATA version 14.0 for analysis. Descriptive statistics such as frequencies, percentages, and cross-tabulations were used to summarize the findings.

**Results:**

The study found that improper sample handling was common during the preanalytical phase. According to the current study, 3.6 % of the sample was rejected at pre analytical stage. The most common reasons for specimen rejection were using inappropriate containers (64.0 %) uncentrifuged specimens (20.4 %); hemolyzed specimens (7.0 %); insufficient specimen volume (6.2 %); clotted specimens (1.9 %); and specimen labeling problems (0.5 %).

**Conclusion:**

This study found that the most common preanalytical error was using an inappropriate sample collection container, followed by uncentrifuged samples, Therefore, it is recommended that mentorship programs be developed to educate staff on the preanalytical phase of laboratory testing, specifically on sample collection, storage, and transportation for HIV viral load testing. Additionally, the quality management system of laboratory processes should be strengthened to ensure accuracy and minimize errors.

## 1 Introduction

### 1.1 Background

Clinical laboratory diagnosis is the cornerstone for healthcare decisions [1], affecting 60–70 % of clinical decisions [2]. Relying merely on clinical symptoms for the diagnosis of diseases can lead to inappropriate treatment, increased patient morbidity, and mortality, and the development of drug resistance [3] (see Table 1).

**Table 1:** Reasons for rejection of HIV viral load specimens referred from August 2021 to November 2022 to Debre Tabor Comprehensive Specialized Hospital, North Central Ethiopia, 2023.

Reason for Specimen Rejection	Number of rejected Specimens	Percent (%) of rejected specimens	Percent (%) of rejected specimens against total collected specimens (n/N)^a^
Inappropriate container	138	64.0	2.5
Uncentrifuged specimens	44	20.4	0.74
Hemolyzed specimens	15	7.0	0.03
Insufficient specimen volume	14	6.2	0.24
Clotted specimens	4	1.9	0.07
Labeling problem of specimens	1	0.5	0.02

a Total number of specimens collected (N) = 5950 and total number of specimens rejected (n) = 216.

The laboratory testing process comprises 3 phases: preanalytical, analytical, and post-analytical [4]. The pre-analytical phase covers all activities and aspects that occurred in different places and times until the sample is ready for testing [2]. It is the laboratory's most error-prone and complicated phase [5,6]. Of the errors that occur during the laboratory testing processes, nearly 68–70 % of errors appear in this phase [1,2,5,7,8].

Appropriate specimen collection, processing, and transportation are crucial to deliver accurate, reliable, and timely results for patients. However, improper collection and handling can lead to repeated tests, client dissatisfaction, and wasting of time and laboratory resources [9]. Poor laboratory results due to improper specimen collection and handling also significantly influence medical diagnosis, affecting proper patient management and outcomes [10]. Accordingly, the reliability of laboratory results depends on the quality of specimen collection and transportation. Therefore, adherence to specimen collection and handling standards is imperative for maintaining specimen integrity and delivering timely and accurate results [10].

If the test cannot be conducted at the collection site or if there are equipment breakdowns, specimens are commonly collected outside the testing laboratory and then transported to testing sites [1], necessitating meticulous processing to preserve their integrity [9]. During transportation, factors such as temperature, preservation methods, specialized transport containers, and storage duration must be carefully managed to ensure the optimal integrity of the sample [9]. Hence, personnel who process the specimens must receive training about proper specimen management. International Organization for Standardization (ISO) recommends that clinical laboratories should establish criteria for accepting or rejecting specimens [9], outlying specific factors that determine whether the specimen is suitable for analysis or not [8]. Therefore, it is critical to ensure that specimens are collected using appropriate containers to maintain their integrity. Standards associated with blood specimen collection, transportation, and handling are available, but compliance with those guidelines is relatively low. The majority of the preanalytical errors arise from issues related to patient preparation, specimen collection, transportation, and preparation for analysis [5].

Monitoring the viral load of human immunodeficiency virus (HIV) in individuals receiving highly active antiretroviral therapy (HAART) is important to ensure the success of the treatment or for deciding on treatment adjustments if needed. Previously, immunological monitoring, particularly (cluster of differentiation (CD)4) counts and clinical assessment were utilized to monitor responses to HAART. However, nowadays the golden standard for monitoring the response of HAART has shifted towards viral load assessment [[11], [12], [13]].

HIV viral load testing requires a plasma specimen obtained from whole blood collected in an ethylene diamine tetra acetic acid (EDTA) test tube. Whole blood is collected typically by phlebotomy, which involves puncturing a vein to draw the blood. Following collection, plasma is separated from whole blood via centrifugation. The plasma can be stored for up to 5 days at a temperature between 2° and 8 °C, and it requires transportation using a cold chain system to maintain its integrity [11]. The plasma HIV viral load indicates the concentration of viral particles per milli liters of specimen. Higher viral load often corresponds to accelerated depletion of CD4 cells and advancement to acquired immunodeficiency syndrome (AIDS). Consequently, viral load assessment has become the preferred method for monitoring HAART response due to its precision and reliability compared to immunological and clinical assessments [11,12].

Viral load specimen rejection and causes for rejections differ among HIV load testing laboratories. Debre Tabor Comprehensive Specialized Hospital Laboratory is one of the testing sites for HIV viral load and the surrounding health facilities send specimens for testing. However, no study has been conducted in the study area to assess the rate and reasons for the rejection of viral load specimens. Thus, this study seeks to address these gaps by investigating the rejection rate and identifying causes for rejection at Debre Tabor Comprehensive Specialized Hospital Laboratory. The findings also assist laboratories in pinpointing areas for improvement to reduce specimen rejection.

## 2 Methodology

### 2.1 Study area

The study was conducted at Debre Tabor Comprehensive Specialized Hospital. The hospital is located in Debre Tabor town at a distance of 103 km in the north-central direction of Bahir Dar, the capital city of Amhara National Regional State. It is also found at a distance of 667 km from Addis Ababa, the capital city of Ethiopia, in the north-central direction. Currently, the hospital provides health services to more than 2.7 million people in different departments. It serves as a comprehensive specialized hospital for the South Gondar Administrative Zone and nearby districts. Moreover, the hospital is the HIV viral load testing site for specimens that are referred from approximately 36 health facilities.

### 2.2 Sample size, study design, and period

A retrospective study was conducted at Debre Tabor Comprehensive Specialized Hospital from January 1st to January 31, 2023. A total of 5950 data were extracted from HIV viral load laboratory specimen tracking log books. These specimens were sent to Debre Tabor Comprehensive Specialized Hospital Laboratory for HIV viral load determination from August 2021 to November 2022.

### 2.3 Rejection criteria set by the laboratory for HIV viral load specimen

Incomplete request.

Sample without request.

Inappropriate transportation.

Misidentification (unlabeled or mislabeled).

Improper container.

Insufficient specimen volume.

Incorrect preservation and storage.

Lipemic specimen.

Uncentrifuged specimen.

Hemolyzed specimen.

Clotted specimens.

Inclusion and exclusion criteria.

The study included referred specimens for HIV viral load determination from August 2021 to November 2022, with all necessary data (specimen accepted or not accepted, if not accepted reason for rejection) correctly recorded in the laboratory HIV viral load specimen tracking logbooks. However, 25 specimens were excluded from the study due to incomplete data (the acceptability or rejection status of the specimens was not known) in the laboratory HIV viral load specimen tracking logbooks.

### 2.4 Study variables

**Outcome variable:** the specimen rejection rate.

**Independent variables:** hemolysis, inadequate volume, clotted specimen, uncentrifuged specimen, mislabeled specimen, inappropriate specimen container.

### 2.5 Data collection tools and technique

Data extraction tools were developed to assess the rate of sample rejection and the causes for the rejection in the preanalytical phase at the Virology Laboratory of Debre Tabor Comprehensive Specialized Hospital. To prepare the data extraction sheet, similar previous works of literature and sample rejection criteria set by Debre Tabor Comprehensive Specialized Hospital Laboratory for HIV viral load specimens were used. Data collectors were given a two-day training on the objectives of the study and about the data extraction tools. Data collectors then reviewed the documents of laboratory specimen tracking log books for the quality of specimens sent from referring sites to the Debre Tabor Comprehensive Specialized Hospital Laboratory for HIV viral load testing, and they extracted the data retrospectively from the HIV viral load laboratory specimen tracking log books.

### 2.6 Data quality assurance and analysis

The data extraction tool was pretested before the commencement of the study to ensure its clarity, acceptability, and consistency to conduct the study, and the necessary corrections were made. The principal investigator reviewed the collected data daily for completeness, clarity, and consistency of the data. The data were entered using double data entry to minimize the occurrence of transcription errors. The data were cleaned and entered into EPI data version 4.6 and transferred to STATA version 14.0 for further analysis. The descriptive statistics were summarized using frequencies, percentages, and cross-tabulations.

### 2.7 Operational definition

**Specimen rejection:** a decision by laboratory personnel that specimens are unacceptable for HIV viral load determination based on the specimen rejection criteria set by the laboratory.

**Specimen rejection rate:** the proportion of rejected specimens among the total number of specimens received from the referring laboratories for HIV viral load determination.

**Insufficient volume:** quantity of specimen less than the minimum needed volume of plasma specimen (1-mL plasma) to conduct HIV viral load determination.

**Hemolysis:** breakdown of red blood cells, which is visible as a reddish color in the clear plasma due to the presence of free hemoglobin.

## 3 Results

In the study period, 5950 specimens were extracted from the laboratory HIV viral load specimen tracking log books. Of these, 216 specimens were rejected because of errors (improper specimen collection and handling) in the preanalytical phase of laboratory diagnosis, accounting for a specimen rejection rate of 3.6 %.

Several factors were documented for the rejection of specimens. Notably, evaluation of the data showed that, of the six reasons for rejection, the most prevalent was using inappropriate specimen containers, 138 (64.0 %) followed by uncentrifuged specimens, 44 (20.4 %), and the last was mislabeled specimens, 1 (0.5 %), as shown in T**able 1**.

## 4 Discussion

Improving the overall quality of laboratory medicine requires recognizing, documenting, and monitoring errors in the laboratory that can compromise the accuracy of test results and patient diagnosis and care [14].

Our research revealed that out of 5950 specimens collected to test HIV viral load for monitoring the effectiveness of antiretroviral drugs, 216 specimens were rejected due to preanalytical errors resulting from poor specimen management. This accounts for a specimen rejection rate of 3.6 %. Since the magnitude of this rejection of the specimen is significant, preventive measures are necessary because improper specimen collection could cause delayed results, incorrect diagnosis, medication errors, and even patient death.

Of the total specimens collected the overall specimen rejection rate found in the current study is similar to the overall specimen rejection rates obtained from studies done at the University of Gondar Hospital, Northwest Ethiopia, 3.8 % [15]; in Zimbabwe, 4 % [16], and Nepal, 5.5 % [17]. However, the overall sample rejection at the pre-analytical phase in the current study is higher than the studies conducted at Amhara Public Health Institute in Bahir Dar, Ethiopia, 0.5 % [9]; St. Paul's Hospital Millennium Medical College in Addis Ababa, Ethiopia, 1.4 % [1]; Delhi, 1.52 % [18]; Cantonal Hospital Zenica, 1.7 % [19]; and Makkah, Saudi Arabia, 1.3 % [20]. On the other hand, our study's finding indicates a lower rate of overall sample rejection in the pre-analytical phase compared to the rate of overall pre-analytical sample rejection of previous studies conducted at St. Paul's Hospital Millennium Medical College in Addis Ababa, Ethiopia,26 % [2]; St. Paul's Hospital Millennium Medical College (SPHMMC) in Addis Ababa, Ethiopia,72.3 % [21], and Saudi Arabia, 12.1 % [14]. This difference could be attributed to the sample size difference, workload, and not adhering to standard specimen collection procedures. To avoid specimen rejection, continuous and on-the-job training for phlebotomists is needed. In addition, communication and specimen collection follow-up procedures should be implemented [9].

According to this study, using inappropriate containers to collect specimens was the most common type of preanalytical error, accounting for 64 % of all types of reasons for sample rejection. This result is higher than the result of a study conducted at Amhara Public Health Institute, Bahir Dar, Ethiopia, which found 17.7 % [9], Cantonal Hospital Zenica, 2.16 % [19], Kenyatta National Hospital, 3.7 % [8] Tertiary Care Hospital in Karad, 7.55 % [22], and Tripoli University Hospital in Libya, 35 % [23] of specimens were rejected at pre-analytical phase due to using of inappropriate containers to collect specimens. This could be due to a lack of refreshment training for phlebotomists. To prevent sample rejection due to using of inappropriate containers to collect specimens, continuous educational updates are needed because healthcare equipment and materials are changed as technology becomes advanced.

The second type of preanalytical error in this study was referring to an uncentrifuged specimen, which accounted for 20.4 % of the total errors. This finding is nearly identical to the result of a study done in Nepal, which found that 20 % of specimens were rejected due to not being centrifuged on a timely base [16]. This result, however, is lower than the study conducted at the Amhara Public Health Institute in Bahir Dar, Ethiopia, at 46.9 % [9]. The reasons for referring an uncentrifuged specimen could be due to a lack of a centrifuge at the referring site or a lack of understanding about the significance of a timely centrifuged specimen.

Another preanalytical error identified in our study was the hemolyzed specimen, which accounted for 7 % of the total errors. This result is higher than the study conducted at Chatrapati Shivaji Hospital, 0.09 % [24]. However, this finding is lower than the result of the study done at the Amhara Public Health Institute in Bahir Dar, Ethiopia, which found that 19.8 % [9]; Oromia, Ethiopia, 11 % [25]; Nepal, 20 % [16]; Turkey, 17.89 % [26]; India, 18.92 % [4]; and Bosnia and Herzegovina, 48.50 % [19] samples were rejected due to hemolysis. This could be due to a lack of standard operating procedures for preventing, detecting, analyzing, and reporting hemolysis. It is important to have the necessary skills, knowledge, and experience to collect a quality specimen that produces accurate results. Poor phlebotomy procedures and not centrifuging the specimen within 4 h could lead to an increase in hemolysis. Additionally, freezing and thawing blood specimens may result in massive hemolysis.

Evidence suggests that a majority of preanalytical errors in laboratories occur due to ineffective specimen collection and management procedures. Among all laboratory errors, the most common cause is collecting unsuitable specimens for testing due to insufficient specimen volume or quantity [27]. Additionally, each analytical process requires a specific volume of serum/plasma for analysis. The primary reasons for this problem could be phlebotomists' lack of skills and difficulty in sampling, such as in pediatric patients, patients with chronic debilitating diseases, and chemotherapy patients with thin and hard-to-locate veins [27]. In our study, 6.2 % of specimens were rejected at the preanalytical stage due to insufficient specimen quantity. This finding is nearly similar to a study conducted at the Cantonal Hospital Zenica in Bosnia and Herzegovina, which found that 11.63 % [19] of samples were rejected due to insufficient quantity. However, this result is higher than the findings of a study conducted at the University of Gondar Hospital in Northwest Ethiopia, 0.1 % [15]; Saudi Arabia, 1.7 % [14]; India, 0.2 % [28]; and India, 1.6 % [27]. On the other side, our study's findings are lower than the result of the study done in Oromia, Ethiopia, 23.3 % [25], and Libya, 50 % [23] of samples were rejected due to insufficient quantity. This could be due to a difference in sample size. The sample size of the Libyan study, for example, was 400. Furthermore, these inconsistencies could be the result of inadequate patient orientation and preparation as well as a poor specimen collection procedure. Therefore, the phlebotomist should orient and prepare the patient before trying to collect the specimen as well as, they should be well informed about the volume of specimens for each type of test.

Blood clotting is most commonly caused by an insufficient anticoagulant-to-blood ratio or a delay in transferring blood from a syringe to a test tube. Clotting damages cells and consumes coagulation factors, rendering the specimen unsuitable for assays that require plasma [20]. In this study, we also discovered another type of preanalytical error (clotted specimen), which accounts for 1.9 % of all errors. This finding is lower than the findings of research conducted in Oromia, Ethiopia, 21.9 % [25]; Saudi Arabia, 20.09 % [20]; Cantonal Hospital Zenica in Bosnia and Herzegovina, 39.87 % [19]; and Libya, 25 % [23] of specimens were rejected due to the occurrence of the clot. A study conducted in Saudi Arabia [14], however, found a lower proportion, i.e., 0.08 % specimen rejection rate due to the clotting of specimens. The general rule is that when the proportion of blood specimens to anticoagulants is high, the specimen will clot. Therefore, to prevent clotting of specimens the ratio of blood to anticoagulant should be appropriate. Furthermore, improper delayed mixing of specimens with anticoagulant after collection causes the occurrence of specimen clotting.

The labeling problem of the specimen was another type of preanalytical problem identified in the current study, accounting for 0.5 % of the total errors. This finding is consistent with the findings of studies conducted in Saudi Arabia,0.5 % [14], and Maryland,0.32 % [29]. However, a higher proportion of specimen rejection due to labeling errors was observed at St. Paul's Hospital Millennium Medical College in Addis Ababa, 16.4 % [30]. Possible explanations for this type of error (specimen rejection due to labeling error) include the phlebotomist's might be inexperience, insufficient on-the-job training about specimen collection and quality, and a heavy workload. To prevent labeling errors, the phlebotomist should label soon after sample collection. In addition, continuous on-the-job training is needed, and assigning newly hired phlebotomists with experienced ones to share skills as well as knowledge.

## 5 Conclusion and recommendation

The findings of the current study provide valuable information that can be used to improve the quality management of laboratory testing and promote better patient care. The most common error was the use of inappropriate specimen collection containers, followed by uncentrifuged specimens. Addressing these errors can help to enhance diagnostic accuracy and achieve better outcomes in laboratory results and patient care. As a result, the findings of this study suggest that mentorship programs on HIV viral load specimen collection, storage, and transportation should be developed. Furthermore, the laboratory process quality management system must be strengthened to identify deficiencies in HIV viral load specimen management. Moreover, it allows for corrective actions to be taken to reduce specimen rejection rates, which have an impact on patient care. Similarly, the frequency of laboratory errors should be monitored and analyzed regularly to identify improvements and patterns that may exist.

## Limitations of the study

The authors review secondary data from laboratory HIV viral load specimen tracking log books. As a result, we were unable to assess the health facilities that sent specimens to Debre Tabor Comprehensive Specialized Hospital's staffing levels and workload. Furthermore, we were unable to verify staff qualifications, competencies, and training for HIV viral load specimen collection. These factors, however, may affect the quality of specimens collected for HIV viral load determination.

## Ethical considerations

This study was reviewed and approved by the research and review committee of the College of Health Sciences, Debre Tabor University with reference No.: CHS/RCC/324/2022. The permission letter was also obtained from Debre Tabor Comprehensive Specialized Hospital to conduct the study. The data were collected retrospectively from HIV viral load specimen tracking log books. Informed consent was not required for this study because the nature of the study design was retrospective. As a result, the research and review committee waived informed consent. Moreover, the data was used solely for the study, and the study was conducted as per the Declaration of Helsinki.

## Consent for publication

Not applicable.

## Funding

There was no funding source for this study.

## Availability of data and materials

The data utilized in our study have not been deposited into a publicly available repository. However, interested parties may request access to the datasets used and/or analyzed during the current study, which are available from the corresponding author, and such requests will be accommodated upon reasonable request.

## CRediT authorship contribution statement

**Ayenew Berhan:** Writing – review & editing, Writing – original draft, Visualization, Methodology, Data curation, Conceptualization. **Andargachew Almaw:** Writing – review & editing, Software, Methodology, Investigation, Formal analysis, Data curation. **Shewaneh Damtie:** Writing – review & editing, Writing – original draft, Software, Formal analysis, Data curation. **Yenealem Solomon:** Writing – review & editing, Writing – original draft, Software. **Biruk Legese:** Writing – review & editing, Software, Investigation, Formal analysis, Data curation. **Birhanu Getie:** Writing – review & editing, Software, Methodology, Formal analysis, Data curation. **Mulat Erkihun:** Writing – review & editing, Software, Methodology, Investigation, Formal analysis, Data curation.

## Declaration of competing interest

The authors declare that they have no known competing financial interests or personal relationships that could have appeared to influence the work reported in this paper.

## Abbreviations

CD: Cluster of Differentiation
HAART: highly active anti-retroviral therapy
HIV: human immunodeficiency virus

## References

[1] Tesfaw H.M., Tsegaye A., Hassen F. (2015). Frequency of specimen rejection and associated factors at st. Paul's hospital Millennium medical College, Addis Ababa Ethiopia. J. Multidiscip. Healthc..

[2] Tadesse H., Desta K., Kinde S., Hassen F., Gize A. (2018). Errors in the hematology laboratory at st. Paul's hospital Millennium medical College, Addis Ababa, Ethiopia. BMC Res. Notes.

[3] World Health Organization (2015).

[4] Jandial S., Gosai V. (2017). Sample rejection rate in clinical biochemistry laboratory of a tertiary care center. Int J Res Med.

[5] Sianipar O. Quality Improvement Attempts in the Preanalytical Phase.

[6] Simundic A.-M., Lippi G. (2012). Preanalytical phase–a continuous challenge for laboratory professionals. Biochem. Med..

[7] Plebani M. (2006). Errors in clinical laboratories or errors in laboratory medicine?. Clin. Chem. Lab. Med..

[8] Felix M.W., Nyagol J., Mwanda W. (2021). Factors associated with sample rejection for CD4+/CD8+ T cell count analyses at the Kenyatta national hospital comprehensive care center laboratory, Kenya. World J. AIDS.

[9] Shiferaw M.B., Yismaw G., Getachew H. (2018). Specimen rejections among referred specimens through referral network to the Amhara Public Health Institute for laboratory testing, Bahir Dar, Ethiopia. BMC Res. Notes.

[10] World Health Organization (2015). Laboratory quality management system (LQMS) training toolkit. External Quality Assessment Module..

[11] National Operational Guidelines for Viral Load Testing http://naco.gov.in/sites/default/files/National%20Operational%20Guidelines%20for%20Viral%20Load%20Testing%20Mar%2718.pdf.

[12] Minchella P.A., Chipungu G., Kim A.A., Sarr A., Ali H., Mwenda R. (2017). Specimen origin, type, and testing laboratory are linked to longer turnaround times for HIV viral load testing in Malawi. PLoS One.

[13] Diress G., Linger M. (2020). Change in viral load count and its predictors among unsuppressed viral load patients receiving an enhanced adherence counseling intervention at three hospitals in northern Ethiopia: an exploratory retrospective follow-up study. JAID (Auckland, NZ).

[14] Alcantara J.C., Alharbi B., Almotairi Y., Alam M.J., Muddathir A.R.M., Alshaghdali K. (2022). Analysis of preanalytical errors in a clinical chemistry laboratory: a 2-year study. J. Med..

[15] Ambachew S., Adane K., Worede A., Melak T., Asmelash D., Damtie S. (2018). Errors in the total testing process in the clinical chemistry laboratory at the University of Gondar Hospital, Northwest Ethiopia. Ethiop. J. Health Sci..

[16] Chiku C., Zolfo M., Senkoro M., Mabhala M., Tweya H., Musasa P. (2019). Common causes of EID sample rejection in Zimbabwe and how to mitigate them. PLoS One.

[17] Pande K., Dahal P., Pokharel L. (2021). Identification of types and frequency of preanalytical errors in hematology laboratory at a tertiary hospital of Nepal. J. Pathol. Nepal.

[18] Chawla R., Goswami B., Tayal D., Mallika V. (2010). Identification of the types of preanalytical errors in the clinical chemistry laboratory: a 1-year study at GB Pant Hospital. J. Lab. Med..

[19] Kadić D., Avdagić-Ismić A., Hasić S. (2019). The prevalence of preanalytical errors in the laboratory of the Cantonal Hospital Zenica in Bosnia and Herzegovina. Med. Glas..

[20] Iqbal M.S., Tabassum A., Arbaeen A.F., Qasem A.H., Elshemi A.G., Almasmoum H. (2023). Preanalytical errors in a hematology laboratory: an experience from a tertiary care center. J. Diagn..

[21] Tadesse H., Desta K., Kinde S., Hassen F., Gize A. (2018 Nov 3). Clinical chemistry laboratory errors at st. Paul's hospital Millennium medical College (SPHMMC), Addis Ababa, Ethiopia. BMC Res. Notes.

[22] Shukla D.K.B., Kanetkar S., Ingale S. (2016). Study of preanalytical and postanalytical errors in hematology laboratory in a tertiary care hospital. J Med Sci Clin Res.

[23] Ashur A, Magrahi H, Bleha Z, Atia A. Assessment of the Preanalytical Phase Errors in the Clinical Laboratory at Tripoli University Hospital.

[24] Upreti S., Upreti S., Bansal R., Jeelani N., Bharat V. (2013). Types and frequency of preanalytical errors in hematology lab. Journal of clinical and diagnostic research. J. Clin. Diagn. Res..

[25] Tola E.K., Dabi Y.T., Dano G.T. (2022). Assessment of types and frequency of errors in diagnostic laboratories among selected hospitals in east Wollega Zone, Oromia, Ethiopia. JPLM.

[26] Keskin A., Aci R., Arslanbek Erdem M., Ari M. (2021). Evaluation of rejection rates and reasons among specimens taken from different hospital units. Med. Lab. J..

[27] Lippi G., Von Meyer A., Cadamuro J., Simundic A.-M. (2019). Blood sample quality. Diagnosis.

[28] Saloni Mittal N. (2020). Study of preanalytical errors in clinical biochemistry laboratory in a rural area of Punjab. Panacea J. Med. Sci..

[29] Rooper L., Carter J., Hargrove J., Hoffmann S., Riedel S. (2017). Targeting rejection: analysis of specimen acceptability and rejection, and framework for identifying interventions in a single tertiary healthcare facility. Clin. Lab. Anal..

[30] Molla H. (2014).

